# The effects of four weeks of creatine supplementation and high-intensity interval training on cardiorespiratory fitness: a randomized controlled trial

**DOI:** 10.1186/1550-2783-6-18

**Published:** 2009-11-12

**Authors:** Jennifer L Graef, Abbie E Smith, Kristina L Kendall, David H Fukuda, Jordan R Moon, Travis W Beck, Joel T Cramer, Jeffrey R Stout

**Affiliations:** 1 Department of Health and Exercise Science, University of Oklahoma, Huston Huffman Center, 1401 Asp Avenue, Norman, OK 73019, USA

## Abstract

**Background:**

High-intensity interval training has been shown to be a time-efficient way to induce physiological adaptations similar to those of traditional endurance training. Creatine supplementation may enhance high-intensity interval training, leading to even greater physiological adaptations. The purpose of this study was to determine the effects of high-intensity interval training (HIIT) and creatine supplementation on cardiorespiratory fitness and endurance performance (maximal oxygen consumption (VO_2PEAK_), time-to-exhaustion (VO_2PEAK_TTE), ventilatory threshold (VT), and total work done (TWD)) in college-aged men.

**Methods:**

Forty-three recreationally active men completed a graded exercise test to determine VO_2PEAK_, VO_2PEAK_TTE, and VT. In addition, participants completed a time to exhaustion (TTE) ride at 110% of the maximum workload reached during the graded exercise test to determine TWD (TTE (sec) × W = J). Following testing, participants were randomly assigned to one of three groups: creatine (creatine citrate) (Cr; *n *= 16), placebo (PL; *n *= 17), or control (*n *= 10) groups. The Cr and PL groups completed four weeks of HIIT prior to post-testing.

**Results:**

Significant improvements in VO_2PEAK _and VO_2PEAK_TTE occurred in both training groups. Only the Cr group significantly improved VT (16% vs. 10% improvement in PL). No changes occurred in TWD in any group.

**Conclusion:**

In conclusion, HIIT is an effective and time-efficient way to improve maximal endurance performance. The addition of Cr improved VT, but did not increase TWD. Therefore, 10 g of Cr per day for five days per week for four weeks does not seem to further augment maximal oxygen consumption, greater than HIIT alone; however, Cr supplementation may improve submaximal exercise performance.

## Background

Traditional endurance training has been shown to improve aerobic capacity, such as the ability to sustain a given submaximal workload for an extended period of time, or to produce a higher average power output over a fixed distance or time [1,2]. Physiological adaptations from training, resulting from an increase in mitochondrial density, include changes in skeletal muscle substrate utilization and improved respiratory control sensitivity [3]. High-intensity interval training (HIIT) is a time-efficient way to induce similar adaptations, such as increased maximal mitochondrial enzyme activity [4] and a reduction in glycogen utilization and lactate accumulation [5,6]. In addition, HIIT may be more effective than conventional endurance training at improving muscle buffering capacity [7,8]. HIIT consists of repeated bouts of short to moderate duration exercise completed at intensities greater than the anaerobic threshold, interspersed with brief periods of low-intensity or passive rest. HIIT is designed to repeatedly stress the body, physiologically, resulting in chronic adaptations and improving metabolic and energy efficiency [9,10]. Helgerud et al. [11] found that HIIT significantly augmented maximal oxygen consumption (VO_2PEAK_) and time to exhaustion (TTE) greater than a traditional training program with moderately-trained males. The velocity at which ventilatory threshold (VT) occurred increased as well, which may signify a higher training capacity and, therefore, should also represent an improvement in endurance performance. It was determined during this study that different protocols of HIIT, matched for frequency and total work done, provided similar results [11]. In support, Burke et al. [12] examined the effects of two different interval training protocols on VO_2PEAK_, VT, and lactate threshold in a group of untrained women, demonstrating that both interval-training protocols significantly improved all performance variables. Similarly, an increase in VO_2PEAK _and VT was found in three groups of well-trained cyclists following three different HIIT protocols of varying intensities and work-to-rest ratios [9].

Phosphocreatine (PCr), a high-energy storage molecule within skeletal muscle, provides immediate replenishment of ATP during intense exercise [13]. Multiple HIIT bouts are designed to deplete PCr stores in the working skeletal muscle, reducing power output. It has been reported that it takes more than six minutes to fully recover depleted PCr stores after exercise-induced PCr depletion [14]. Therefore, if recovery intervals during HIIT bouts are less than six minutes, PCr may not be fully replenished, resulting in a reduced ability to meet the demands of cellular ATP resynthesis and a reduced performance [13]. Supplementing with creatine (Cr) has been demonstrated to effectively augment muscle phosphocreatine (PCr) stores [15]. Specifically, one study showed a 20% increase in muscle creatine with ingestion of 20 g of Cr per day for just 5 days [16].

It has been suggested that increases in skeletal muscle PCr concentration may improve muscle buffering capacity and moderate glycolysis [17,18]. In addition, Cr supplementation may increase the rate of PCr resynthesis between HIIT exercise bouts and enhance mitochondrial shuttling of ATP into the cytoplasm, providing significant physiological adaptations [15,16].

Current research suggests that Cr supplementation, when combined with training, has been shown to significantly augment performance [19]. Moreover, the combination of Cr supplementation and HIIT may lead to greater improvements in VO_2PEAK_, VT, and TTE than previously reported with HIIT or Cr supplementation alone. While Cr is known to improve anaerobic performance, its use in aerobic performance has been under-researched. To date, no one has examined the concurrent effects of Cr supplementation and HIIT. Therefore, we propose that Cr supplementation may increase training capacity during HIIT, resulting in improved endurance performance as measured by VO_2PEAK_, VT, and TTE, beyond what has been demonstrated for HIIT alone. The purpose of this study was to determine the combined effects of four weeks of HIIT and Cr supplementation on VO_2PEAK_, VT, and TTE in recreationally active college-aged men.

## Methods

Forty-three recreationally active (1-5 hours of regimented exercise per week) college-aged men (mean ± SD, Age: 22.6 ± 4.9 years; Ht: 178.1 ± 7.1 cm; Wt: 83.0 ± 13.8 kg) volunteered to participate in this study. Participants were screened for health conditions that would have prevented them from participating in the study, including heart and joint conditions. Any participants who had taken sports supplements, including any form of Creatine, in the three months prior to the beginning of the study were excluded. Participants kept a food diary, and none of the participants consumed a vegetarian diet. Participants were asked not to change training or dietary habits for the duration of the study. This study was approved by the University's Institutional Review Board for Human Subjects and informed consent was obtained from each participant prior to enrollment.

### Determination of VO_2PEAK_, ventilatory threshold, and total work done

A maximal graded exercise test (GXT) on a cycle ergometer (Corival 400, Groningen, The Netherlands) was completed by all participants to determine maximal oxygen consumption (VO_2PEAK_). Participants began pedaling at a cadence of 60-80 revolutions per minute (RPM) at a workload of 20 watts (W). The workload increased 1 W every 3 seconds (a total of 20 W every minute). This continued until the subject could no longer maintain 60-80 RPM or until volitional exhaustion, despite verbal encouragement. Respiratory gases were monitored and continuously analyzed with open-circuit spirometry using a calibrated metabolic cart (True One 2400^®^, Parvo-Medics, Inc., Provo, UT). Data were averaged over 15-second intervals, with the highest 15-second oxygen consumption and heart rate recorded as the peak oxygen consumption (VO_2PEAK_) and maximum heart rate (HRmax), respectively. Time to exhaustion for the GXT (VO_2PEAK_TTE) was recorded. In addition, ventilatory threshold (VT) was measured during this test. VT was determined from a plot of ventilation (V_E_) against VO_2 _as described previously [20]. Two linear regression lines were fit to the lower and upper portions of the V_E _vs. VO_2 _curve, before and after the break points, respectively. The intersection of these two lines was defined as VT.

At least 24-48 hours following the VO_2PEAK _test, participants returned to determine time to exhaustion (TTE) at 110% of the maximum workload reached during the graded exercise test. Participants completed a warm-up consisting of a five-minute cycle at a workload of 50 W. Following warm-up, participants pedaled at 110% of the maximum workload achieved during their VO_2PEAK _test. Keeping a cadence of 70 RPM, they pedaled until volitional exhaustion. Time was recorded in seconds, and total work done (TWD) was reported in kilojoules, determined by multiplying the workload in watts and the time to exhaustion in seconds.

Reliability of VO_2PEAK_, VT and TWD was determined using a subsample of subjects (n = 10) measured during each scheduled testing week. The test-retest intraclass correlation coefficient (*R*) was 0.96 (SE ± 0.1 L), 0.67 (SE ± 0.3 L), and 0.79 (SE ± 4.8 kJ), respectively, for the three measurement variables.

A total of three testing sessions occurred throughout a nine-week period--familiarization (week 1), baseline (week 4), and post (week 9). Familiarization testing was implemented to reduce any learning effect--possibly influencing the dependent variables as well as the training intensity--from the initial VO_2PEAK _testing.

### Supplementation

Following familiarization testing, participants were randomly assigned, in a double-blind fashion to either a Cr (n = 16) or a Pl (n = 17) group. A control group (CON; n = 10), neither supplemented nor completed the high-intensity interval training, and instead only completed the testing measurements during each of the scheduled testing weeks. Participants supplemented for a total of 30 days (10 days of familiarization period followed by an additional 20 days of supplementing and training) at a dose of 10 g per day, taken in two doses--one dose 30 minutes prior to and one dose immediately following training. Participants only supplemented on training days (5 days/week) under the supervision of the researchers, to monitor compliance. Participants in the Cr group consumed 5 g of creatine citrate mixed with 15 g dextrose per packet (Creatine Edge, FSI Nutrition, Omaha, NE), dissolved in 4-8 ounces of water. Similarly, participants in the PL group consumed 20 g of dextrose per packet dissolved in 4-8 ounces of water. Both drinks were identical in appearance and taste.

### High-intensity interval training (HIIT)

**Figure 1:**
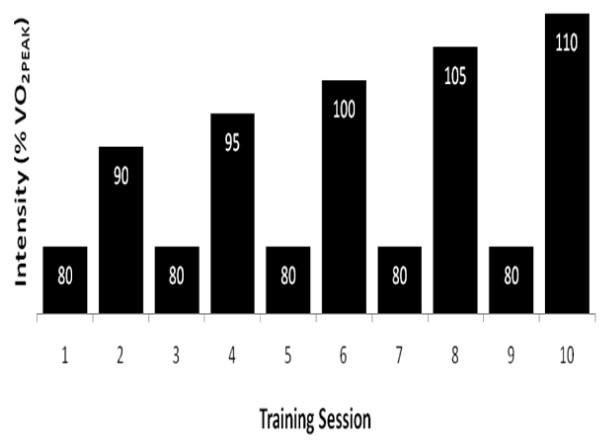
**HIIT protocol**. Represents the first two weeks of the HIIT protocol. Training intensity eventually reached 120% of the VO_2PEAK _maximum workload.

Training began at least 24-48 hours following the TTE test. Participants were required to visit the lab five days per week, for six weeks, to perform the HIIT. A two-week familiarization training period was implemented before taking baseline testing measurements. Due to the effectiveness of the training, and to the generally untrained population, a familiarization period was implemented to allow for all participants to quickly adapt to the high-intensity protocol. Previous research has shown significant improvements in performance with just two weeks of HIIT [21]. Furthermore, in a previous study from our lab in which a familiarization period was not used, the large adaptations from training may have masked any effects from supplementation [22]. After the two-week familiarization period, participants re-tested their VO_2PEAK _and TTE. Following baseline testing, participants completed four additional weeks of training, in which the intensities were re-evaluated based on baseline VO_2PEAK _power output values. Three of the five days per week of training consisted of training at progressively increasing workloads, determined as a percentage of the participant's baseline VO_2PEAK _max workload. One recovery day (two days per week) occurred between each of the three difficult training sessions. During these recovery days, participants completed a training session at 80% of their VO_2PEAK _max workload. Difficult training days increased in intensity each session beginning at 90% of their VO_2PEAK _max workload and progressing up to 120% of their VO_2PEAK _max workload (Figure 1). Each training session began with a five-minute warm up at 50 W, followed by a protocol of five sets of two-minute exercise bouts, with one minute of passive rest in between exercise bouts.

### Statistical analysis

Descriptive statistics were evaluated to determine group demographics. A mixed factorial ANOVA (group [Cr vs. Pl vs. Con] × time [pre vs. post]) was evaluated, looking for any significant differences (*P *≤ 0.05) between treatment groups and across time for each variable measured. If a significant interaction occurred, the statistical model was decomposed and the simple main effects were examined using separate one-way repeated measures ANOVAs for each group. If the result was a simple main effect, Bonferroni *post-hoc *comparisons were performed among groups, while dependent-samples t-tests with Bonferroni corrections were performed across time. If no interactions occurred, the main effects were analyzed by collapsing across the non-interacting variables and analyzed in the same approach as described for the simple main effect.

## Results

Separate one-way ANOVAs indicated no differences between groups in any of the variables at baseline measurement. In addition, there was no change measured in the Con group over time in any of the variables.

### Body Weight (BW)

There was no change in BW from baseline to post measurement in the Cr (84.0 ± 12.5 kg and 84.4 ± 12.3 kg, respectively) or Pl (82.9 ± 15.2 kg and 83.2 ± 15.0 kg, respectively) groups.

### Maximal Oxygen Consumption (VO_2PEAK_) and Time to Exhaustion (VO_2PEAK_TTE)

A significant two-way interaction (time × treatment, p < 0.001) for VO_2PEAK _occurred, and a post hoc Bonferroni analysis indicated no significant differences between groups at post measurements. However, a main effect for time (p < 0.001) occurred due to a change in VO_2PEAK _over time in the Cr (p = 0.002) and Pl (p = 0.001) groups, as indicated by separate Bonferroni-adjusted (p < 0.017) dependent-samples t-tests (Table 1).

**Table 1:** Mean ± SD of maximal oxygen consumption (VO_2PEAK_), time to exhaustion (VO_2PEAK_TTE), and ventilatory threshold (VT) at baseline and following four weeks of treatment

	**VO**_**2PEAK **_**(l·min^**-1**^)**		**VO**_**2PEAK**_**TTE (seconds)**		VT (l·min^**-1**^)	
	**Baseline**	**Post**	**Baseline**	**Post**	**Baseline**	**Post**

**Cr (n = 16)**	3.60 ± 0.55	3.87 ± 0.47*	818.3 ± 127.2	869.3 ± 130.0*	2.14 ± 0.53	2.49 ± 0.57*
**Pl (n = 17)**	3.65 ± 0.59	4.00 ± 0.59*	837.7 ± 130.1	899.4 ± 127.9*	2.30 ± 0.51	2.54 ± 0.48
**Con (n = 10)**	3.67 ± 0.71	3.54 ± 0.71	802.8 ± 148.9	781.9 ± 151.2	2.08 ± 0.70	1.99 ± 0.48

*Indicates a significant (p ≤ 0.01) change over time within treatment groups.

There was a significant two-way interaction (time × treatment, p < 0.001) for VO_2PEAK_TTE; however, a post hoc Bonferroni analysis indicated no significant differences between groups at post measurement. A main effect for time (p < 0.001) occurred, and separate Bonferroni-adjusted (p < 0.017) dependent-samples t-tests indicated a significant change over time in the Cr (p < 0.001) and Pl (p < 0.001) groups.

### Ventilatory Threshold (VT)

**Figure 2:**
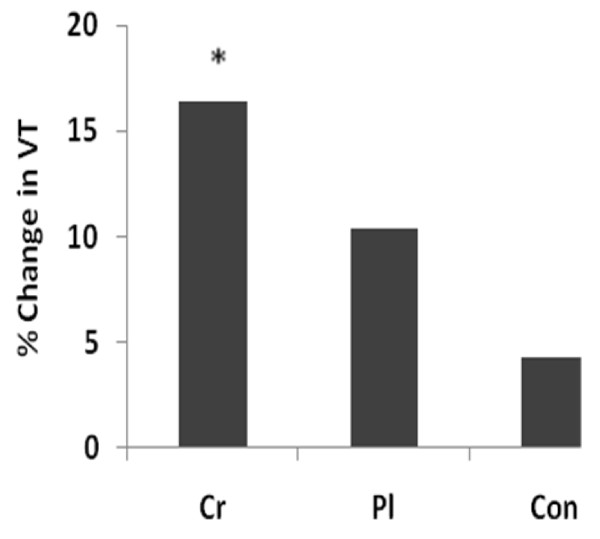
**Effect of Creatine and HIIT on VT**. Percent change in VT over time for each group.

A significant two-way interaction (time × treatment, p = 0.040) occurred for VT (l·min^-1^). A post hoc Bonferroni analysis indicated no difference between Cr and Pl (Table 1). Separate Bonferroni-adjusted (p < 0.017) dependent-samples t-test indicated a change over time for Cr (p = 0.001), but not for Pl (p = 0.040) (Figure 2).

### Total Work Done (TWD)

Table 2 summarizes the mean changes in TWD at 110% of the VO_2PEAK _maximum workload within the three treatment groups. There was no interaction and no main effect for time for either group.

**Table 2:** Mean ± SD of total work done (TWD) at 110% of VO_2PEAK _maximum workload at baseline and following four weeks of treatment

	TWD (kJ)	
	**Baseline**	**Post**

**Cr (n = 16)**	42.3 ± 8.0	40.5 ± 9.4
**Pl (n = 17)**	47.5 ± 14.1	43.3 ± 10.0
**Con (n = 10)**	37.7 ± 9.1	39.0 ± 11.6

## Discussion

High-intensity interval training has been shown to be an effective method for improving endurance performance [7,12,23-26]. The results of the present study are in agreement with many studies demonstrating an increase in VO_2PEAK _after HIIT [12,27-29]. In addition, time to exhaustion during the graded exercise test was also improved. However, few studies have examined the concurrent effects of HIIT with Cr supplementation on endurance performance. The current study demonstrated no additional improvements in VO_2PEAK _when combining Cr supplementation and HIIT. However, when measuring VT, improvements were only demonstrated in the Cr group. Interestingly, in contrast to previous reports of significant increases in TWD with Cr supplementation or HIIT alone, no change in TWD was observed [5,28,30-33].

Endurance performance is commonly assessed using a measure of aerobic capacity, VO_2PEAK_. HIIT has been reported to be effective in improving VO_2PEAK _5-15% [12,27-29,34-40]. In the current study, a 9% increase in VO_2PEAK _was observed. Short recovery periods between intense exercise bouts during HIIT may create a greater reliance on aerobic metabolism, due to the importance of aerobic metabolism in the removal of lactic acid and for the resynthesis of phosphocreatine [41]. HIIT may also induce up-regulation of glycolytic and oxidative enzymes, a possible mechanism influencing the improvements in VO_2PEAK _[34]. In addition, an increase in stroke volume following HIIT [11] may contribute to an increase in VO_2PEAK_.

While the HIIT program was effective in improving VO_2PEAK _by 9%, creatine supplementation had no further influence on aerobic capacity. These results are in agreement with the few studies that have examined the effects of Cr supplementation on VO_2PEAK _[30,42-44]. Cr has been shown to be effective in improving short-duration, intense activities, but few studies have examined the effects of Cr on longer duration, endurance-type activities. Due to the intensity and time duration (two minutes) of the interval work periods, it was hypothesized that Cr would provide for a greater training capacity, and, therefore, the Cr group would show greater improvements in the testing measurements. McConell and colleagues [45] found that Cr improved the maintenance of energy balance in the muscle during intense aerobic exercise; however, performance was not improved, which is in agreement with the current study.

Ventilatory threshold (VT) may be another useful predictor of endurance performance. The VT has been suggested as an indicator of the ability of the cardiovascular system to adequately supply oxygen to the working muscles, preventing muscle anaerobisis [46]. Performing exercise at intensities greater than VT commonly result in an inadequate supply of oxygen to the working muscles, quickly leading to fatigue [47]. Therefore, improvements in VT may correspond to an augmented time to exhaustion and a greater threshold for fatigue. Additionally, it has been proposed that training at intensities greater than VT, much like the HIIT protocol of the current study, may enhance the efficiency of the body to supply oxygen to the working muscles (i.e. VT) [12,48-50]. Furthermore, a concomitant rise in muscle lactate levels and a drop in pH at high intensities of exercise may signal arterial chemoreceptors, altering ventilatory regulating mechanisms. Therefore, improvements in cardiovascular fitness may also coincide with a decrease in lactate accumulation resulting in an improvement in VT. However, in the current study, significant improvements in VT were only observed in the Cr group (16%), although the Pl group demonstrated a trend for improved VT (10%). The increased VT in the Cr group is in agreement with previous studies that demonstrated improved VT following Cr supplementation but without training [30,42,44]. Improved exercise efficiency at submaximal workloads following Cr supplementation may be attributed to an increase in muscle PCr levels, which may augment the ratio of ATP/ADP, stimulating mitochondrial respiration [42] and delaying reliance on anaerobic glycolysis [51,52]. In addition to acting as an energy buffer, PCr also acts as a proton (H^+^) buffer when the creatine kinase reaction favors regenerating ATP [53]. This utilization of H^+ ^may delay the decrease in skeletal muscle pH, possibly signaling arterial chemoreceptors and augmenting the ventilatory response. Future studies, however, are needed to validate our results.

Both HIIT and Cr have been reported to improve total work done [5,28,30-33]. However, no improvements were observed in TWD during a ride to exhaustion at 110% of the maximum workload reached during the GXT in the present study. One reason for the lack of improvement in TWD in the current study may be participant motivation. The 110% workload during which TWD was measured was the first ride to exhaustion in a session of 3 rides to exhaustion. Therefore, participants may have quit early in order to save energy for the subsequent work bouts. In addition, studies that observed improvements in TWD following Cr supplementation implemented a loading phase (20 g/d for 5-7 days) into the supplementation protocol [30-33]. A loading phase was not used in the current study, so it may be possible that muscle PCr levels were not increased enough to aid in improving TWD.

## Conclusion

In conclusion, the current study supports previous evidence that HIIT is an efficient way to induce cardiorespiratory improvements [7,12,23-26]. However, although Cr supplementation has been shown to improve intense exercise [54,55], no apparent benefits were observed in the present study. Furthermore, while improvements in VT were observed following Cr supplementation, it did not lead to an increase in TWD. A Cr loading phase followed by a maintenance phase might improve HIIT more than the low-dose supplementation used in the current study. However, Jager et al. found improvements in interval exercise performance using a similar dose of creatine citrate (5 g/day for 28 days) [56]. Due to the possibility that any benefits of low-dose creatine supplementation were masked by the effectiveness of HIIT alone, a longer training period may be implemented in future studies to determine whether low-dose Cr supplementation will induce further improvements when results from training begin to plateau.

## Competing interests

The authors declare that they have no competing interests.

## Authors' contributions

JG, AS, KK and DF contributed in writing and editing the manuscript along with concept and design, data acquisition, and data analysis and interpretation. JM, TB, JC, and JS contributed in writing and editing the manuscript, as well as concept and design. All authors have read and approved the final manuscript.
